# 8-Methoxypeucedanin: Evaluation of Anxiolytic Effects and Modulation of Neuronal Activity Related Genes in a Zebrafish Anxiety Mode

**DOI:** 10.3390/ijms262110259

**Published:** 2025-10-22

**Authors:** Jarosław Widelski, Monika Maciąg, Natalia Kasica, Barbara Budzyńska, Piotr Podlasz, Simon Vlad Luca, Dafina Fondai, Krystyna Skalicka-Woźniak

**Affiliations:** 1 Department of Pharmacognosy with Medicinal Plant Unit, Medical University of Lublin, 20-093 Lublin, Poland; 2 Independent Laboratory of Behavioral Studies, Medical University of Lublin, 20-093 Lublin, Poland; monika.maciag@umlub.pl (M.M.); barbara.budzynska@umlub.pl (B.B.); 3 Department of Animal Anatomy, Faculty of Veterinary Medicine, University of Warmia and Mazury in Olsztyn, 10-719 Olsztyn, Poland; natalia.kasica@uwm.edu.pl; 4 Department of Pathophysiology, Forensic Veterinary Medicine and Administration, Faculty of Veterinary Medicine, University of Warmia and Mazury in Olsztyn, 10-719 Olsztyn, Poland; piotr.polasz@uwm.edu.pl; 5 Institut de Chimie Organique et Analytique, Université d’Orléans, CNRS, UMR 7311, BP6759, 45067 Orléans, France; simon-vlad.luca@univ-orleans.fr; 6 Department of Pharmacy, Faculty of Medicine, University of Prishtina, 10000 Prishtina, Kosovo; dafina.fondaj1@gmail.com; 7 Department of Chemistry of Natural Products, Medical University of Lublin, 20-093 Lublin, Poland; kskalicka@pharmacognosy.org

**Keywords:** furanocoumarins, anxiety, *bdnf*, *c-fos*, neural activity, *Danio rerio*, thigmotaxis

## Abstract

For thousands of years, medicinal plants and their constituents have been used, mostly empirically/ethnopharmacologically, to cure patients with central nervous system (CNS) disorders. Anxiolytics derived from natural products (NPs) often share similar mechanisms of action to synthetic ones (e.g., benzodiazepines, BDZs). Although typically as effective as synthetic anxiolytics, NPs are considered to be devoid of the serious side effects linked to the use of BDZs. 8-Methoxypeucedanin (8-MP) is a rare furanocoumarin present in the fruits of *Peucedanum luxurians* Tamamsch. (Apiaceae). The primary objective of the presented study was to assess the anxiolytic activity of 8-MP using a zebrafish (*Danio rerio*) model of anxiety. *Danio rerio* larvae at 5 days post-fertilization (dpf) were used, with reversed thigmotaxis considered as an index of the anxiolytic activity. In addition to the behavioral study, qPCR analyses were performed to assess the role of 8-MP in modulating the expression of *c-fos* and *bdnf*, two key genes involved in neural activity. As evidenced by the behavioral study, 8-MP (1.5–15 µM) exhibited a significant influence on anxiety, with a U-shape dose–response effect. Moreover, the expression of *c-fos* and *bdnf* genes was significantly downregulated, providing novel insights into the mechanisms of action of the tested furanocoumarin.

## 1. Introduction

Anxiety disorders are defined as a more prolonged state of tension, worries, and apprehension regarding often undefined, potentially negative, and future events [1]. In the present time, anxiety-related diseases are the most predominant neuropsychiatric disorders, both in Europe and the United States. According to popular and widespread opinion, they are characteristic of well-developed societies in modern times [2].

Among all mental diseases, anxiety disorders, including panic disorder, generalized anxiety disorder (GAD), social anxiety disorder (SAD), different types of phobias, and separation anxiety disorder, are the best characterized [3]. The severity associated with a variety of anxiety disorders is aggravated by unsatisfied pharmacotherapy.

Benzodiazepines (BDZs) remain the first-line and most effective choice in the treatment of anxiety disorders [4]. Drugs belonging to this therapeutic class (e.g., diazepam) are γ-aminobutyric acid (GABA)-enhancers, acting by increasing the affinity and binding of neurotransmitter (GABA) at the GABA_A_ receptors. As a consequence, the neurotransmission of the neurons of the limbic system (especially the amygdala) is inhibited [5]. Despite their high efficacy and the fact that their short-term use is well tolerated, BDZs have a number of serious side effects, such as dizziness, concentration disturbances, and memory impairment [6]. Severe disorders are associated with the long-term use of BDZs and include the development of tolerance, physical dependence, and withdrawal syndrome [7]. Thus, searching for new alternative substances to be used in the management of anxiety and related disorders is an urgent and crucial task.

Among numerous classes of phytochemicals, coumarins have attracted great interest in the last few years due to their versatile and readily accessible scaffold with broad-ranging biological activities [8]. The more than 1300 known natural coumarins are generally divided into the following groups: simple coumarins, isocoumarins, biscoumarins, furanocoumarins, and pyranocoumarins (the last two categories subdivided into angular and linear) [8,9]. Their interesting structural and physico-chemical features (e.g., low molecular weight, relatively simple structures, high solubility in most organic solvents, lipophilicity) ensure their role as leading compounds in drug research and development [8,10,11]. Among the numerous biological activities reported for natural coumarins, those concerning the influence on the central nervous system (CNS) are of particular interest. A large number of coumarin derivatives have been reported to exhibit potent CNS effects, as assessed through in vitro and in vivo platforms. These include modulation of GABA, dopamine, and serotonin neurotransmission, inhibition of acetylcholinesterase (AChE), butyrylcholinesterase (BChE), monoaminoxidases A and B (MAO-A, MAO-B), and neuroprotection [12]. Thus, coumarins can be regarded as potential therapeutic drug lead candidates in the management of many psychiatric and neurodegenerative diseases, like anxiety and related disorders, depression, epilepsy, and even Alzheimer’s disease.

The majority of anxiety-related behavioral tests that evaluated the effects of natural coumarins on CNS were performed on rodents (mostly mice and rats) and were based on the internal conflict of the animal between the need to explore a new environment and avoidance of the open space [13]. The most frequently used test for the rodent model of anxiety is the elevated plus maze (EPM), where the indicator of the anxiolytic activity of the tested substances is the time spent in the open arms (i.e., an increase in this parameter typically confirms the tested activity) [14]. Nowadays, the *Danio rerio* (zebrafish) model can be used for high-throughput screening of anxiolytic activity, in addition to widely used rodent models [13]. The zebrafish shares a lot of similarities with vertebrates in the field of genetics, brain pattering, structure, and function in neurochemical and behavioral systems. What is noteworthy is that the general organization and function of their stress-regulating system have are highly analogous with mammals [15,16]. Another advantage of the zebrafish model is the fact that animals can be easily monitored using automated behavioral tracking software, significantly enhancing the efficiency of the test and reducing interrater variance [17].

Last but not least, zebrafish (including larvae) are highly sensitive to psychotropic drugs (such as anxiolytics), and their pharmacological effects are relatively easy to observe and measure [17,18]. Furthermore, numerous studies confirmed the feasibility of implementing rodent behavioral paradigms in zebrafish [15]. The anxiety evaluation indicates similar effects in both *Danio rerio* and rodent models [13]. Anxiety-related behaviors can be evaluated by measuring thigmotaxis, which includes a preference for peripheral areas (outer zone or border zone) and avoidance of the center of the open area (central zone) [19]. Reduction in thigmotaxis (i.e., thigmotactic behavior) is directly related to the anxiolytic effect of the test substance.

**Figure 1:**
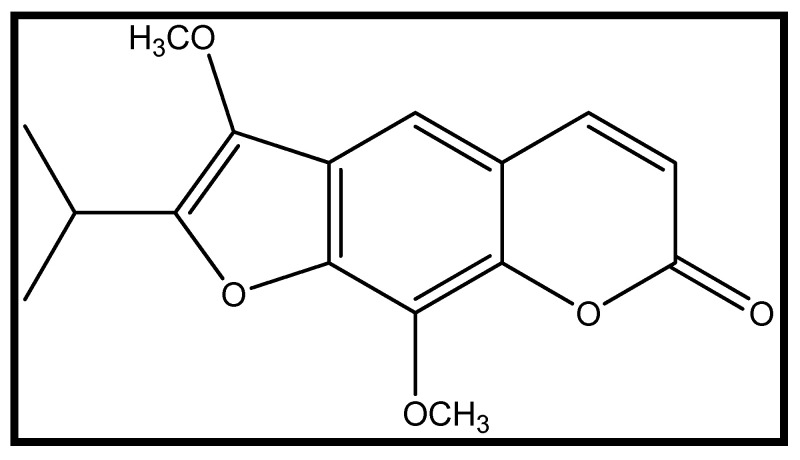
Structure of 8-methoxypeucedanin (8-MP).

8-Methoxypeucedanin (8-MP, Figure 1) is a rare furanocoumarin present in the fruits of *Peucedanum luxurians* Tamamsch. (Apiaceae). 8-MP has been previously isolated by our group with high purity and in sufficient amounts for biological testing by countercurrent chromatography [20]. Our previous research on *P. luxurians* revealed their immunomodulating [21] and antibacterial activities [20]. Nevertheless, representatives of the *Peucedanum* genus are abundant in coumarins with different chemical scaffolds responsible for numerous biological activities, in particular, CNS effects [22]. For instance, ostruthin, imperatorin, ostruthol, and oxypeucedanin hydrate from *P. ostruthium* (L.) Koch showed AChE-inhibitory activity [23]; bergamottin and lucidafuranocoumarin A isolated from *P. alsaticum* L. showed moderate inhibition activity against AChE and BChE activity. Moreover, lucidafuranocoumarin A was found to be a significant inhibitor of PTZ (pentylenetetrazole)-induced seizures in vivo [24]. In addition, the existing literature highlights the anxiolytic activity of other furanocoumarins in models of rodents [25] and zebrafish [13], providing a rationale for testing 8-MP towards this direction. Thus, the aim of this study was to evaluate the anxiolytic potential of 8-MP in a zebrafish model on 5 days post-fertilization (dpf) larvae. Furthermore, in order to provide links into the potential mechanisms of action, the influence of 8-MP on the expression of selected genes involved in neural activity (i.e., *c-fos* and *bdnf*) was assessed.

Our study focuses on evaluating the anxiolytic potential of 8-methoxypeucedanin (8-MP), a rare furanocoumarin, using 5-day-old zebrafish larvae as a high-throughput in vivo model. The innovation lies in investigating a previously unexplored compound for its behavioral effects on anxiety, complemented by an analysis of neural activity-related gene expression (c-fos and bdnf). Unlike previous research that mostly relied on rodent models, our approach leverages the advantages of zebrafish larvae, including their sensitivity to psychotropic substances, ease of behavioral tracking, and conserved stress-regulating neurocircuitry. This integrated behavioral and molecular assessment provides novel insights into both the efficacy and potential mechanisms underlying the anxiolytic effects of 8-MP.

## 2. Results

### 2.1. Evaluation of the Anxiolytic Activity

#### 2.1.1. The Influence of 8-MP on the Spontaneous Locomotor Activity of *Danio rerio* Larvae

**Figure 2:**
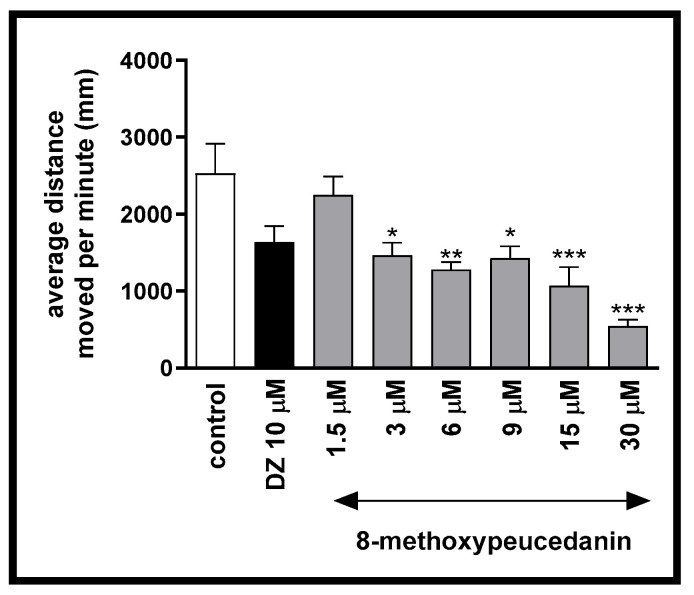
The effects of 8-methoxypeucedanin (1.5, 3, 6, 9, 15, 30 µM) and diazepam (10 µM) on locomotor activity during 40 min light phase. Average distance (mm) moved by zebrafish larvae within each 1 min time bin. Data are presented as mean ± SEM, n = 32. * *p* < 0.05, ** *p* < 0.01, *** *p* < 0.001 in comparison to the control group, post hoc Tukey’s test.

One-way ANOVA showed that 8-MP has an influence on the locomotor activity of *Danio rerio* larvae at 5-dpf: [F (7, 250) = 11.41, *p* < 0.001; Figure 1]. Post hoc Tukey’s test confirmed that 8-MP decreased the locomotor activity of larvae at concentrations of 3 µM (*p* < 0.05), 6 µM (*p* < 0.01), 9 µM (*p* < 0.05), 15 µM (*p* < 0.001), and 30 µM (*p* < 0.001) (Figure 2).

#### 2.1.2. The Influence of 8-MP on Thigmotactic Behaviors of the *Danio rerio* Larvae

**Figure 3:**
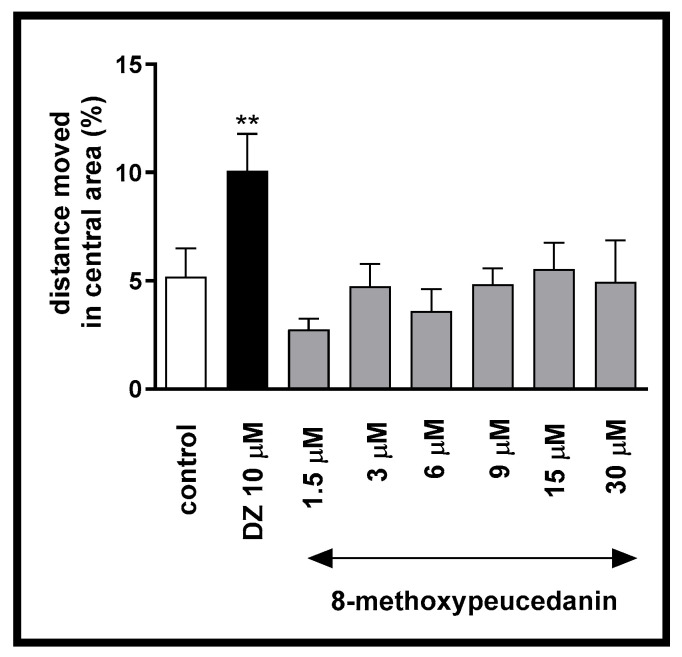
The effects of 8-methoxypeucedanin (1.5, 3, 6, 9, 15, 30 µM) and diazepam (10 µM) on the percentage of the distance moved in the central arena under continuous illumination. Data are presented as mean ± SEM, n = 32, ** *p* < 0.01 in comparison to the control group, post hoc Tukey’s test.

One-way ANOVA showed statistically significant changes induced by 8-MP when considering the distance moved in the inner zone during 40 min of continuous light [F (7, 250) = 2.835, *p* = 0.0177; Figure 3]. Post hoc Tukey’s test confirmed that diazepam increased the percentage of distance moved (*p* < 0.01) in the central arena in comparison to the DMSO-treated group (control group).

**Figure 4:**
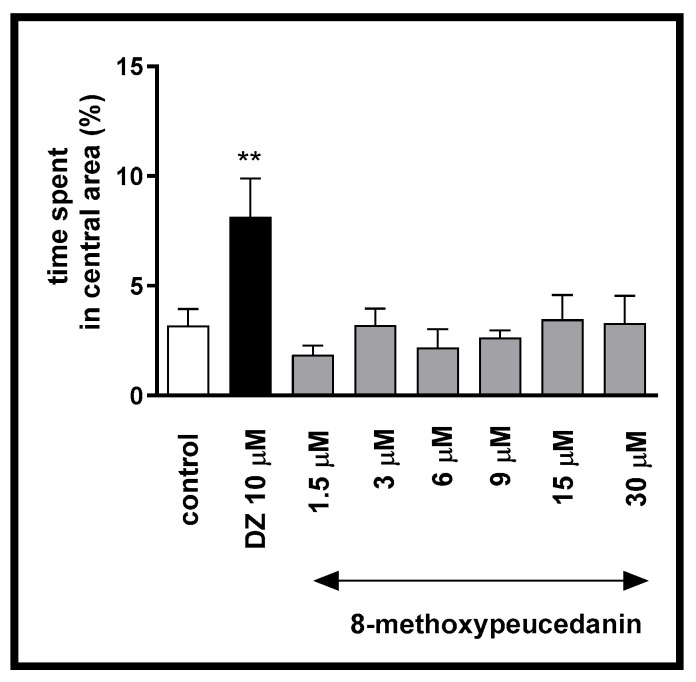
The effects of 8-methoxypeucedanin (1.5, 3, 6, 9, 15, 30 µM) and diazepam (10 µM) on the percentage of the time spent in the central arena under continuous illumination. Data are presented as mean ± SEM, n = 32, ** *p* < 0.01 in comparison to the control group, post hoc Tukey’s test.

In the case of 8-MP, one-way ANOVA also showed a statistically significant difference in the time spent in the inner zone during 40 min of continuous light [F (7, 250) = 3.972, *p* = 0.0025; Figure 4]. The post hoc test (Tukey’s) confirmed that this parameter (time) was increased by diazepam (*p* < 0.01) in comparison to the control group (DMSO-treated) (Figure 4).

#### 2.1.3. The Influence of 8-MP on the Locomotor Activity of *Danio rerio* Larvae During the Light–Dark Challenge

**Figure 5:**
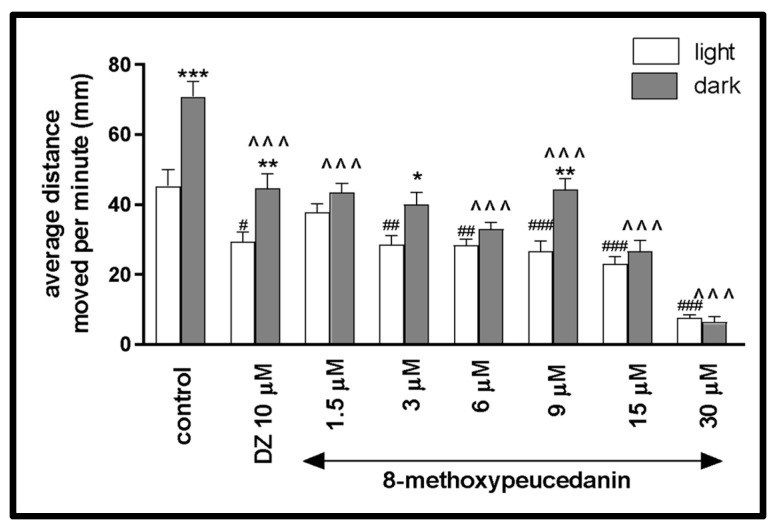
The influence of 8-methoxypeucedanin (1.5, 3, 6, 9, 15, 30 µM) on the locomotor activity during all three light–dark challenge phases. The average distance moved by the zebrafish larvae within each 1 min during the light phase (white bars) or dark phase (filled bars) was plotted. Data are presented as mean ± SEM. * *p* < 0.05, ** *p* < 0.01, *** *p* < 0.001 in comparison to the light conditions within the same concentration group; ^^^ *p* < 0.001 in comparison to the control group under dark condition (post hoc Bonferroni’s test); # *p* < 0.05, ## *p* < 0.01, ### *p* < 0.001 in comparison to control group under light condition (post hoc Bonferroni’s test).

The average distance traveled by the larvae during the light–dark challenge (all three cycles) was measured for better characterization of the effect of the tested coumarin on the locomotor activity during this phase of the experiment. For 8-MP, the two-way ANOVA showed statistically significant changes in the zebrafish larvae behavior in light–dark condition [F (1, 236) = 15.34, *p* = 0.0001], treatment effect [F (7, 236) = 51.90, *p* < 0.0001], as well as interaction [F (7, 236) = 6.615, *p* < 0.0001]. The post hoc Bonferroni’s test showed an increase in the locomotor activity during the dark phase in the control group (DMSO, *p* < 0.001), diazepam (*p* < 0.01), and 8-MP at the concentrations of 3 µM (*p* < 0.05) and 9 µM (*p* < 0.01), in comparison with the light phase (Figure 5). Decreases in the locomotor activity during the dark phase were observed after incubation with diazepam (*p* < 0.001) and 8-MP at concentrations of 1.5 µM (*p* < 0.001), 6 µM (*p* < 0.001), 9 µM (*p* < 0.001), 15 µM (*p* < 0.001), and 30 µM (*p* < 0.001), in comparison with DMSO-treated group in the dark phase (Figure 5). During the light phase of the experiment, significant changes in the locomotor activity were noticed after incubation with 8-MP at concentrations of 1.5 µM (*p* < 0.01) and 30 µM (*p* < 0.05) when compared to the DMSO-treated group in the light phase (Figure 5).

#### 2.1.4. The Influence of 8-MP on the Thigmotactic Behavior of *Danio rerio* Larvae During the Light–Dark Challenge

**Figure 6:**
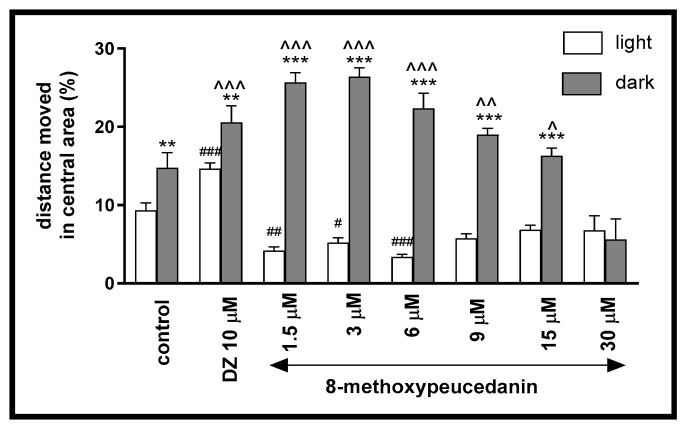
The influence of 8-methoxypeucedanin (1.5, 3, 6, 9, 15, 30 µM) and diazepam (DZ, 10 µM) on the percentage of the distance moved by zebrafish larvae in the central area during the light phase (white bars) or dark phase (gray bars). Data are presented as mean ± SEM; n = 32, ** *p* < 0.01, *** *p* < 0.001 in comparison to the light conditions within the same concentration group; ^ *p* < 0.05, ^^ *p* < 0.01, ^^^ *p* < 0.001 in comparison to the control group under dark condition; # *p* < 0.05, ## *p* < 0.01, ### *p* < 0.001 in comparison to control group under light condition (post hoc Bonferroni’s test).

Treatment with 8-MP during the light/dark challenge had an impact on the thigmotactic behavior of *Danio rerio* larvae when the distance traveled in the central arena was considered (two-way ANOVA: interaction [F (7, 334) = 29.48, *p* < 0.0001], light–dark condition [F (1, 334) = 312 3, *p* < 0.0001], treatment [F (7, 334) = 11.93, *p* < 0.0001]. Post hoc Bonferroni’s test revealed an increase in the percentage of the distance traveled by the 5-dpf larvae in the central arena (inner zone) after incubation with the control group (*p* < 0.01), diazepam (*p* < 0.01), and 8-MP at concentrations of 1.5, 3, 6, 9, and 15 µM (*p* < 0.001) in the dark phase, as compared with the light phase. Furthermore, during the dark phase of the experiment, the distance traveled in the central area increased significantly when 5-dpf larvae were treated with diazepam (*p* < 0.001) and 8-MP at concentrations of 1.5, 3, 6 µM (*p* < 0.001), 9 µM (*p* < 0.01), and 15 µM (*p* < 0.05), as compared to the control group (DMSO-treated) in the dark phase (Figure 6). During the light phase of the experiment, a significant increase in the percentage of the distance moved in the central arena was noticed when 5-dpf larvae were treated with diazepam (*p* < 0.001). However, a decrease was induced by 8-MP at concentrations of 1.5 µM (*p* < 0.01), 3 µM (*p* < 0.05), and 6 µM (*p* < 0.001), when compared to the DMSO-treated group in the light phase (Figure 6).

**Figure 7:**
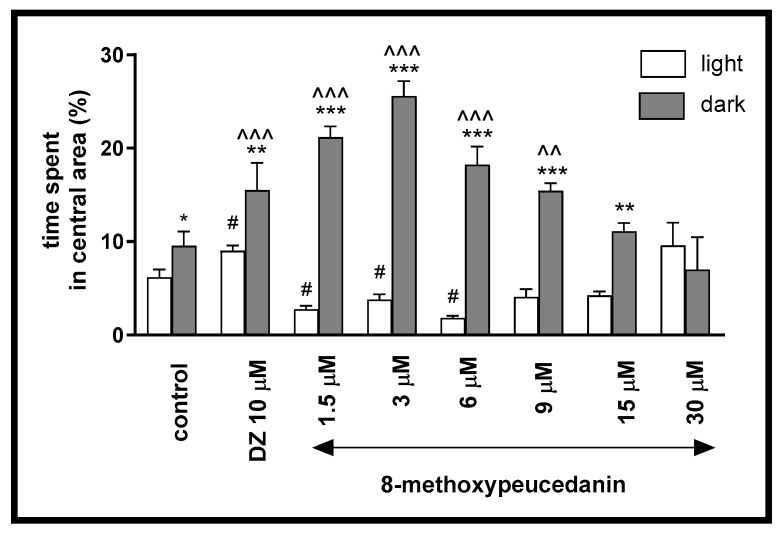
The influence of 8-methoxypeucedanin (1.5, 3, 6, 9, 15, 30 µM) and diazepam (DZ, 10 µM) on the percentage of the distance moved by zebrafish larvae in the central area during the light phase (white bars) or dark phase (gray bars). Data are presented as mean ± SEM; n = 32. * *p* < 0.05, ** *p* < 0.01, *** *p* < 0.001 in comparison to light conditions within the same concentration group; ^^ *p* < 0.01, ^^^ *p* < 0.001 in comparison to the control group under dark condition; # *p* < 0.05, in comparison to the control group under light condition (post hoc Bonferroni’s test).

At the tested concentrations, 8-MP reduced thigmotaxis (suggesting an anxiolytic activity). This effect is indicated by two-way ANOVA analysis (treatment F (7, 334) = 6.847, *p* < 0.0001, interaction [F (7, 334) = 20.71, *p* < 0.0001], light–dark condition [F (1, 334) = 174.2, *p* < 0.0001]. Post hoc Bonferroni’s test revealed an increase in the percentage of the time spent by the 5-dpf larvae in the central arena (inner zone) after incubation with DMSO (control, *p* < 0.05), diazepam (*p* < 0.01), and 8-MP at concentrations of 1.5, 3, 6, 9 µM (*p* < 0.001), and 15 µM (*p* < 0.01) in the dark phase, as compared with the light phase (Figure 7). In addition, during the dark phase of the experiment, significant increases in the time spent in the central area were observed when the 5-dpf larvae were treated with diazepam (*p* < 0.001) and 8-M at concentrations of 1.5, 3, 6 µM (*p* < 0.001), and 9 µM (*p* < 0.01), as compared with the control group (DMSO-treated) in dark phase (Figure 7). During the light phase of the experiment, a significant increase in the percentage of the time spent in the central arena was noticed in the case of diazepam (*p* < 0.05). The same parameter decreased at concentrations of 8-MP of 1.5, 3, and 6 µM (*p* < 0.05) when compared to the DMSO-treated group in the light phase (Figure 7).

### 2.2. Influence of 8-MP on the Expression of Genes Associated with Neural Activity

The effect of diazepam (10 µM) and 8-MP (6 µM) on the level of expression of two genes associated with neural activity (i.e., c-fos and *bdnf*) was determined by RT-qPCR. A dose of 6 µM was chosen for 8-MP based on the results of the anxiolytic activity tests (behavioral tests). The influence of diazepam and 8-MP on *bdnf* gene expression in the 5-dpf zebrafish larvae was also evaluated. They were treated with tested substances and subjected to behavioral tests involving the triggering of anxiety through the use of variable lighting (transition light to dark).

**Figure 8:**
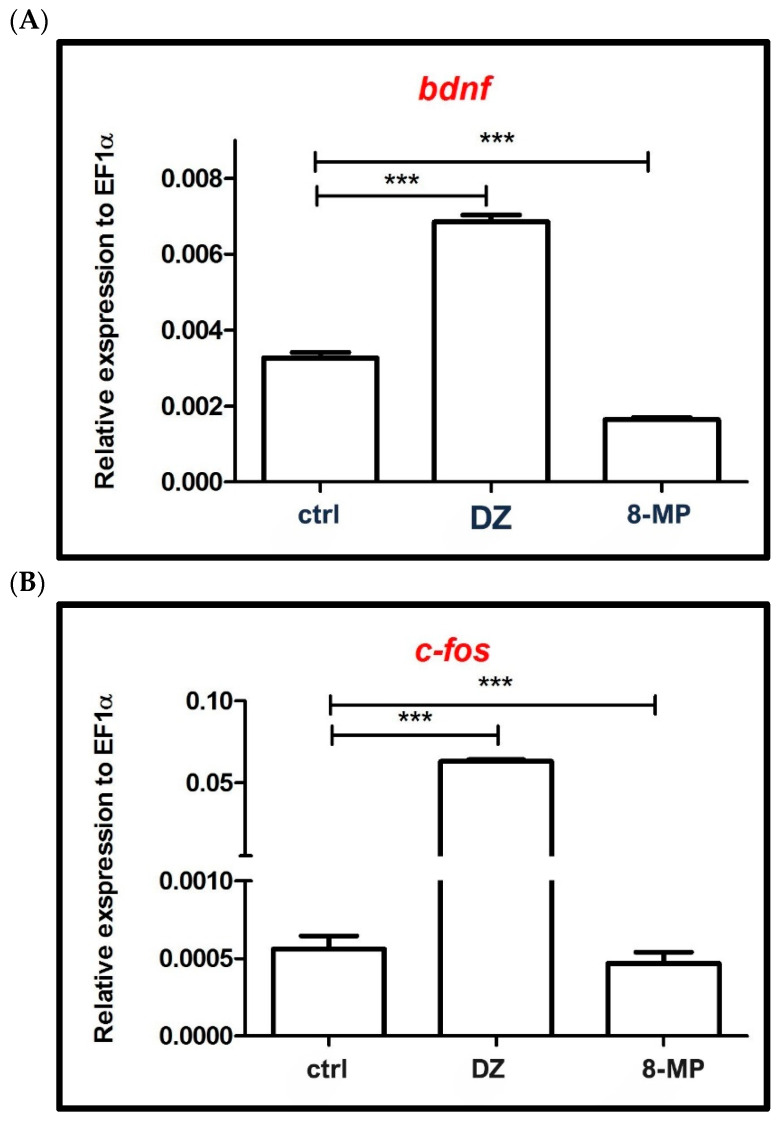
Expression profiles of *bdnf* (**A**) and *c-fos* (**B**) genes after exposure to diazepam (DZ, 10 µM) and 8-methoxypeucedanin (6 µM). Data are presented as mean ± SEM; *** *p* < 0.001 in comparison to the control (ctrl) group.

In compliance with Figure 8, the acute administration of 8-MP and diazepam had a significant influence on the expression of the *bdnf* gene. In contrast to diazepam, which significantly increased the level of *bdnf* expression (*p* < 0.001), the tested furanocoumarin decreased its level of expression (*p* < 0.001) in the zebrafish larvae compared to the control group. The same trend was observed with respect to *c-fos* gene expression. 8-MP decreased the *c-fos* gene expression level (*p* < 0.001), while diazepam increased its expression (*p* < 0.001) in the zebrafish larvae compared to the control group (Figure 8).

## 3. Discussion

For the first time, the effect of 8-MP, a rare furanocoumarin, on anxiety-related behavior in the zebrafish model of anxiety was investigated. The utility of the zebrafish model for appraising the influence of compounds of natural origin with a well-known murine paradigm was previously confirmed by Maciąg and colleagues [13]. Our results indicate that 8-MP has a pronounced anxiolytic activity in *Danio rerio* larvae. A bell-shaped dose effect was noticed. The anxiolytic effect was observed at medium-tested doses; at a higher dose, it decreased; finally, an anxiogenic effect at a concentration of 30 µM (not statistically significant) was noticed. 8-MP at doses of 1.5, 3, 6, and 9 µM increased the time spent and distance moved in the inner (central) zone during the light/dark transition. It is noteworthy to mention that the tested furanocoumarin showed stronger activity than diazepam. The doses of the applied substance were even 6-fold lower. During the first phase of the experiment (under continuous light), 8-MP did not show an influence on the spontaneous locomotor activity. Hence, we can suggest that the observed anxiolytic effect was not connected to a modulation of the locomotor activity of larvae. Later, during the light/dark phase of the experiment, all concentrations of 8-MP (except the highest dose, 30 µM), as well as the control and diazepam groups, increased the percentage of the time spent and distance moved in the inner zone in the dark phase in comparison to the light phase. Moreover, during the dark phase of the light/dark transition, 8-MP increased both parameters, i.e., the time (spent) and distance (moved) in the inner zone, when compared to the control group in the dark phase, displaying an anxiolytic action.

Our research showed that acute administration of 8-MP induces anxiolytic activity, which decreases gradually with increasing the dose of tested coumarin. The trend was also noticed for other coumarins, such as xanthotoxin [13] and imperatorin [25].

Data obtained, both in experimental and clinical studies, have postulated that the stimulation of GABAergic neurotransmission induces an anticonvulsant effect and decreases the anxiety level (i.e., exhibits anxiolytic effect) [26]. Moreover, it was observed that xanthotoxin (substitution of methoxy group in the position C-8 of psoralen ring) enhanced the anticonvulsant activity of carbamazepine and valproate (both possessing described behavioral effect through GABAergic mechanism) [27]. It is probable that the presence of non-polar moiety in position C-8 of the psoralen scaffold is crucial for the GABAergic activity of furanocoumarins (and their anticonvulsant and anxiolytic activity) [12]. This structure–activity relationship is confirmed for imperatorin (with C-8 isoprenyl-ether substituent) and xanthotoxin (methoxy group) [28]. Similarly, 8-MP possesses a methoxy group at the same position.

Although there is no data in the literature confirming the anxiolytic effect of 8-MP through its influence on GABAergic neurotransmission, this mechanism of action could be suggested for 8-MP. This hypothesis is made based on the chemical features and analogies of 8-MP with other coumarins with confirmed pharmacological activity (substitution with a non-polar group at position C-8; linear type of furanocoumarins) [27].

Another possible mechanism (except affinity to the BZD site of the GABA_A_ receptor) of anxiolytic activity through GABA transmission depends on the inhibition of GABA-T (GABA transaminase), an enzyme responsible for GABA degradation in the brain. A decrease in the GABA level leads to hyperactivity, which is linked to the development of many mental disorders, including anxiety and epilepsy [29]. Moreover, several anticonvulsants that do not act directly on GABA_A_ receptors are considered promising anxiolytic agents (e.g., vigabatrin, gabapentin) [30]. Also, few studies have discovered the influence of medicinal plants with anxiolytic activity on the major enzymes of the GABA system (especially GABA-T) [30]. The effect of increasing inhibitory GABAergic transmission based on a dual mechanism of action involving both receptor affinity (IC_50_ = 8.0 ± 0.8 µM) [31] and inhibition of GABA breakdown (second-order rate constant—*k_inact_* = 2.3 ± 0.2 mM^−1^min^−1^) was proven for imperatorin [32]. A similar activity towards the GABA system was suggested for xanthotoxin due to its similarity in chemical structure to imperatorin (both are 8-C derivatives of psoralen) [13].

Based on the literature data and the results of the study, it can be hypothesized that the anxiolytic activity of 8-MP results from a molecular mechanism of activity similar to other C-8 substituted furanocoumarins. The more potent anxiolytic effect observed for 8-MP in the current study in comparison to xanthotoxin [13] in the zebrafish model can be linked to structural differences between the tested compounds. It is likely that the non-polar substituents in the structure of 8-MP (i.e., a second methoxy group at C-11 and isoprenyl moiety at C-12 on the psoralen scaffold) increase their lipophilicity and, hence, the permeation of the blood–brain barrier, thus, improving the anxiolytic activity of the tested furanocoumarin [33]. It is worth mentioning that 8-MP at the higher dose exerted an anxiogenic effect. This tendency was also observed for another C-8 derivative of psoralen (i.e., xanthotoxin) [13], and it could be induced by the modulation of monoamines neurotransmission (e.g., serotonin and catecholamines: dopamine, adrenaline, and noradrenaline) [34]. Furthermore, modulators of monoamine neurotransmission exert pleiotropic effects on mental and cognitive functions, i.e., induce anxiety. As a matter of fact, it was revealed that enhanced noradrenergic transmission is associated with anxiety [35,36]. Moreover, several animal studies proved that the anxiety state is connected with an increase in the release of noradrenaline, especially in the hypothalamus [37], leading to the activation of the hypothalamic–pituitary–adrenal axis, thereby eliciting anxiety [38,39]. In turn, altered serotoninergic and dopaminergic [40] activity can be responsible for either anxiolytic or anxiogenic effects, and the evidence remains inconclusive [41].

Furthermore, we may also suggest that a bell-shaped curve of anxiolytic activity of 8-MP can be the consequence of the metabolism of coumarin and its time-dependent distribution [42].

Even based on obtained behavioral results and knowledge about the mechanism of anxiety and neurotransmitter circuits, we can, with high probability, suggest enhancement of the level of GABA and influence on GABA_A_ receptor as a mechanism underlying the anxiolytic activity of the tested coumarin [43]. Nevertheless, the diversity of coumarin structure, the complexity level of molecular processes (especially in the CNS), and the used model of anxiety (with some differences concerning enzyme and neurotransmitter systems between zebrafish and other animals) are the indicators that the exact mechanism of anxiolytic activity is still to be discovered.

Additionally, in the frame of our studies on the anxiolytic activity of 8-methoxy coumarins, the influence of the expression level of two genes (*c-fos* and *bdnf*) connected with neuronal activity was evaluated. The tested furanocoumarin downregulated (*p* < 0.001) the relative expression of zebrafish *c-fos* and *bdnf* expression. Contrary effects were observed for diazepam, which significantly upregulated (*p* < 0.001) the relative expression of both genes. In adult zebrafish, the light/dark test stimulated *c-fos* activation in particular brain regions connected to stress reactions, like the stress center of the hypothalamus and dorsal telencephalon, which is a zebrafish homolog of the mammalian amygdala [44,45]. In studies conducted by Chen and co-workers [45], the expression level of the *c-fos* gene was significantly increased in the hypothalamus of fish larvae that underwent the light/dark choice behavior. At the same time, lorazepam (benzodiazepine) treatment alleviated the increased *c-fos* expression. Moreover, control animals were characterized by low to moderate levels of *c-fos* mRNA in the forebrain and low levels of *c-fos* mRNA in the hypothalamic regions [45]. Compared to the control, during the light/dark choice box challenge, zebrafish exerted increases in *c-fos* gene expression. Treatment with lorazepam significantly decreased hypothalamic activity and restored *c-fos* expression to the level of the control group [45]. Those results correspond with the one obtained in our study concerning the influence of 8-MP on the relative expression of *c-fos* genes. It seems that the anxiolytic effect of the tested furanocoumarin is connected to the suppression of neuronal activity. The light/dark environment likely exerted sustained mild stress to provoke the anxiety-related state in the zebrafish larvae, while the benzodiazepines (lorazepam) can attenuate the anxious state by reducing the neural activity in broad regions of the brain [45].

In other studies, Almeida and co-workers showed that PTZ-induced seizures increased the *c-fos* transcript levels, which were symptoms of neural excitability [46]. Diazepam was not able to prevent the increase in the *c-fos* transcript levels [46], which is similar to the result of our studies.

Brain-derived neurotrophic factor (BDNF) is broadly expressed in the adult mammalian brain as well as its receptor tyrosine receptor kinase B (TrkB) [47,48]. In humans and rodents, decreasing hippocampal BDNF concentrations may be involved in the onset of depression and anxiety, concerning the fact that the hippocampus, as a part of the limbic system, is considered to be engaged in learning, mood, and anxiety [48].

In another study, Maffioli et al. [49] proved that the reduced expression of BDNF apparently exerts an anxiolytic-like effect on fish behavior by lowering the freezing events, meandering values, time spent at the bottom of the tank, and time spent in the dark compartment. Thus, the influence of compounds with anxiolytic activity, especially furanocoumarins, on the expression of *bdnf* should be studied further.

## 4. Materials and Methods

### 4.1. Chemicals

The following compounds were used during experiments: diazepam (at concentration 10 µM, Sigma-Aldrich, St. Louis, MO, USA), 8-methoxypeucedanin [3,9-dimethoxy-2-propan-2-ylfuro [3,2-g]chromen-7-one, 8-MP]. The tested furanocoumarin was isolated from the fruits of *Peucedanum luxurians* Tamamsh. (Apiaceae) according to previously used procedures [20]. The plant material (mature fruits) was obtained from the Botanical Garden of the University of Adam Mickiewicz in Poznań, Poland (voucher specimen: 7973_S003).

### 4.2. Animals

The maintenance and breeding of zebrafish (*Danio rerio*, AB strain) followed standard laboratory procedures described previously [50]. Adult fish were kept in a recirculating aquaculture system at 28.5 °C under a 14 h light/10 h dark photoperiod. Fertilized eggs were obtained by natural spawning and transferred to Petri dishes containing E3 embryo medium (pH 7.1–7.3; 17.4 µM NaCl, 0.21 µM KCl, 0.12 µM MgSO_4_). Embryos were incubated at 28.5 °C under the same light regime using an IN 110 incubator (Memmert GmbH, Büchenbach, Germany). Behavioral tests were carried out on 5 days-post-fertilization (dpf) larvae. After testing, larvae were euthanized by immersion in tricaine solution (15 µM). All experimental procedures conformed to the EU Directive 2010/63/EU and the NIH guidelines for the care and use of laboratory animals. Ethical approval was not required for experiments using zebrafish larvae up to 5dpf.

#### 4.2.1. Zebrafish Experiment

The anxiolytic-like behavior assay was carried out using 5-day-old zebrafish larvae placed individually in the wells of a 24-well plate (one larva per well). A diazepam stock solution (100 mM in DMSO) was diluted with E3 medium to obtain a 10 µM working solution. The test compound, 8-methoxypsoralen (8-MP), was first dissolved in DMSO at a concentration of 50 mM and subsequently diluted to the desired working concentrations with E3 medium. For each treatment, 1.5 mL of the corresponding solution was added to the wells. The control group received E3 medium containing 1% DMSO. The concentration range for 8-MP (1.5, 3, 6, 9, 15, and 30 µM) was selected based on results from preliminary experiments [50]. Larvae were exposed to each concentration for 30 min prior to behavioral testing.

#### 4.2.2. Anxiolytic Activity Assessment

Behavioral recordings were performed using a Zebrabox system (Viewpoint, Lyon, France) equipped with ZebraLab software (https://www.viewpoint.fr/product/zebrafish/fish-behavior-monitoring/zebralab, accessed on 16 October 2025) for automated video tracking. The experimental protocol followed the general procedure described by Schnorr et al. [19]. Each trial lasted 95 min and consisted of an initial 10 min acclimation period, followed by 40 min of continuous illumination to assess spontaneous locomotor activity. Subsequently, larvae were subjected to three alternating light–dark cycles (15 min each), consisting of 10 min of light followed by 5 min of darkness, to evaluate anxiety-like responses. For analysis of thigmotaxis, each well was virtually divided into two zones: an outer perimeter and an inner (central) zone. The software calculated the time spent and distance traveled in the central zone relative to the total arena area. Results were expressed as the percentage of time and distance recorded in the inner zone.

### 4.3. RNA Isolation and Quantitative PCR

Immediately after exposure to fear-inducing conditions (light–dark transition), larvae of each group were sampled (n = 30) and frozen (−80 °C). Total RNA was extracted using the Total RNA Mini Isolation Kit (AA Biotechnology, Gdynia, Poland) according to the manufacturer’s protocols. cDNA samples required for qPCR analyses were synthesized from matrix samples with normalized RNA concentration using the Maxima First Strand cDNA Synthesis Kit for RT-qPCR (Thermo Scientific, Waltham, MA, USA). Reverse transcription was performed according to the manufacturer’s instructions. qPCR was performed using SYBR Green (SYBR Select Master Mix, Applied Biosystems, Foster City, CA, USA) on a 7500 Fast Real-Time PCR System instrument (Applied Biosystems, Foster City, CA, USA) under previously described conditions [51]. Oligonucleotide primers selected to detect genes involved in neuronal activity are listed in Table 1. Initial validation of the reference genes showed that, for the purpose of the study, elongation factor 1-alpha (ef1-alpha) showed the most efficient and consistent expression among the samples. The expression values of the investigated genes in each group were calculated as relative expression to Ef1-alpha. Each sample was analyzed in triplicate in three separate experiments.

### 4.4. Statistical Analysis

Statistical analyses were performed using Prism software (The last one version) (https://www.graphpad.com/features, GraphPad Software, San Diego, CA, USA). Results are presented as the mean ± standard error of the mean (SEM). Differences between treatment groups and concentrations were evaluated using one-way or two-way analyses of variance (ANOVA), depending on the experimental design. When significant effects were detected, Tukey’s multiple comparison test (for one-way ANOVA) or Bonferroni’s post hoc test (for two-way ANOVA) was applied. Statistical significance was accepted at a confidence level of *p* < 0.05.

## 5. Conclusions

The aim of our present study was to evaluate the anxiolytic activity of 8-MP, a rare furanocoumarin isolated from *Peucedanum luxurians* Tamamsch. This work is the first attempt to assess its activity towards CNS, using a zebrafish model of anxiety based on 5-dpf larvae. The obtained results showed a great potency of this furanocoumarin derivative and its significant influence on anxiety levels, more potent than the activity of diazepam, an anxiolytic drug from BDZs. The research mentioned above also proves the utility of the zebrafish model as a tool for high-throughput screening of the activity of natural products, in particular, coumarins. The model appears to be an efficient and predictive platform for behavioral research of substances obtained from natural resources and facilitation in understanding the structure–activity relations of evaluated compounds. Nevertheless, confirmation of the activity of coumarins from *Peucedanum luxurians* on other models of anxiety and further experiments for the purpose of understanding the mechanism of activity and possible side effects are necessary.

## Figures and Tables

**Table 1:** Primers used in the study.

*Gene*	Forward 5′-3′	Reverse 5′-3′	Accession No/Source
*c-fos*	GTGCAGCACGGCTTCACCGA	TTGAGCTGCGCCGTTGGAGG	NM_205569.1
*bdnf*	GGCGAAGAGCGGACGAATATC	AAGGAGACCATTCAGCAGGACAG	NM_131595.2
*Ef1α*	CTGGAGGCCAGCTCAAACAT	ATCAAGAAGAGTAGTACCGCTAGCATTAC	NM_131263.1

## Data Availability

Raw data are available at Medical University of Lublin (Experimental Medicine Center–OMD).
